# Supplementary material for: In-memory mechanical computing
Source: Nat Commun. 2023 Aug 25;14:5204. doi: 10.1038/s41467-023-40989-1 (PMC10457397; doi:10.1038/s41467-023-40989-1)
Supplement: Supplementary file 1 — Supplementary Information [file 41467_2023_40989_MOESM1_ESM.pdf]

Supplementary information for

In-memory mechanical computing

Tie Mei, Chang Qing Chen *

*Corresponding author. Email: chencq@tsinghua.edu.cn (C.Q.C.)

Supplementary Note 1: Additional computing process of the mechanical XNOR structure

In the main text, the computing process of the mechanical XNOR structure has been introduced for the input being (0, 0) and (1, 0). Note that the computing process for the input being (0, 1) is the same as that of (1, 0) owing to the upper-bottom symmetry of the design structure. Here, we will only discuss the state change when the input is (1,1), as shown in Supplementary Figure 1. If the input is (1, 1), the two input buckled beams compress the two spring 1 simultaneously (Supplementary Figure 1a). Considering that the geometry of the input buckled beams is the same, the compressive force at both ends of the balance bar 1 is also the same, and balance bar 1 remains vertical. Thus, the slider bars and links stay at their initial position. When the structure is subject to a time signal (external force *F*), the common vertex of the two link 1 will push the output buckled beam for a distance of Δ (Supplementary Figure 1b). Driven by link 2 and link 3, two of the balance bar 2 rotate and touch the slider bars. The right ends of spring 1 are also pushed for a distance of Δ' and the input buckled beams are initialized to state 0. Considering the output buckled beam arching to the right, its state will switch to 1 after the force *F* is released, i.e., the structure outputs 1 when the inputs are (1, 1).

Supplementary Note 2: Assembling of the basic mechanical interaction structures

The assembling details of the basic mechanical interaction structures are shown in Supplementary Figure 3. For the mechanical shift register structure (Supplementary Figure 3a), slider block 1 is fixed into slide groove 1 and can only move translationally along the *x* direction. Link 2 (marked in Fig. 2), slider block 2, and slider block 3 are fixed into slider groove 2 and can only move translationally along the *y* direction. The ends of the buckled beam are inserted into the slot and are fixed. To impose the time signal (external force *F* shown in Fig. 2b and c), the electromagnet will be connected to the connecting block (Supplementary Figure 3b). Driven by the electromagnet, the connecting block moves downward and the slider block 1 moves towards each other. Thus, the computing can be done as shown in Fig. 2.

For the mechanical XNOR structure (Supplementary Figure 3c), slider block 1, link 2 in Fig. 3, and slider block 2 are fixed into the slider groove 1, 2, and 3, respectively. Thus, slider 1 can only move translationally along the *x* direction while link 2 and slider block 2 can only move translationally along the *y* direction. The balance bar 1 and 2

(marked in Fig. 3) are connected to the support via cylinder 1 and 2. They can only rotate around these cylinders. Besides, the slider block marked in Fig. 3 is connected to link 2 by the insert block. It follows the motion of link 2.
The assembling details of the mechanical perceptron structure are shown in Supplementary Figure 3d. Slider block 1, 2, and 3 are fixed into the slider groove 1, 2, and 3, respectively. All these slider blocks can only move translationally along the y direction.

Supplementary Note 3: Design of other structures required for computing

Designs of several other typical structures required to facilitate computing are given in Supplementary Figure 4. For the mechanical binary neural network shown in Fig. 4, bias is stored in the structure illustrated in Supplementary Figure 4a. If the buckled beam is in state 0 (Supplementary Figure 4ai), the rubber band makes the links point to the positive direction of the y axis. Thus, the motion of the likes is fixed by the support when an external force F is imposed. However, if the buckled beam is in state 1 (Supplementary Figure 4aii), the links will generate a displacement load Δ driven by F. Note that the state of the memory unit in this structure will not be influenced by the motion of components. Thus, this structure can be used for long-term memory of the bias.

Moreover, every input mechanical memory unit in the first layer influences two memory units in the second layer as shown in Fig. 4e. Thus, we can design a variant one for the mechanical XNOR operation (Supplementary Figure 4b), where the input buckled beam is connected to two springs and can drive two XNOR operations at the same time. Besides, for long-term memory of the weights, the balance bar and slider block are shortened. Thus, they cannot initialize the input buckled beams to state 0 as shown in Fig. 3d and Supplementary Figure 1b.

For the mechanical self-learning perceptron shown in Fig. 5, we have designed a data selector to facilitate the computing process (Supplementary Figure 4c). The data selector consists of one controlling bar and two input bars (Supplementary Figure 4ci). There are two blocks on the controlling bar (see the sectional view in Supplementary Figure 4cii). The controlling bar can selectively block the movement of the input bars by these blocks to realize the function of the data selector shown in Fig.5b. Inputs of

the data selector In1 and In2 are defined as the movement of the input bars 1 and 2, while the output is defined as the movement of the block at the right of the input bars (Supplementary Figure 4ciii and civ). If c=0, block 2 will block the movement of input bar 2, thus the output will be In1 (Supplementary Figure 4ciii). As for c=1, the movement of input bar 1 will be blocked by block 1, thus the output will be In2 (Supplementary Figure 4civ). Thereby, the structure fulfills the requirements of a data selector mentioned in Fig. 5b.

Supplementary Note 4: Training of the binary neural network and construction of mechanical binary neural network with different network structures

To get the weight and bias of a mechanical binary neural network (BNN), a corresponding binary neural network should be trained first. We adopted the binary neural network model proposed in Ref 43 of the main text. The BNN is trained with two sequential runs of the Stochastic Gradient Descent (SGD) procedure. In the first round, the binary weight and bias are transferred to the real weight and bias by the hyperbolic tangent function, $\tanh(\cdot)$ which is also used for activation. In doing so, a relaxed version of the corresponding BNN is obtained and trained. In the second round, the weight and bias in the first round are binarized to initialize the BNN parameters. A noisy feed-forward step is followed, and the parameters are updated while letting the network be aware of the additional error introduced by the binarization procedure. By repeating these two rounds, the BNN is trained.

To verify the training method, a BNN is trained to distinguish between labeled images of handwritten digits. 50 images of each digit from the MINST database (Ref 44) are picked randomly as the training set while additional 50 images of each digit serve as the testing set. Each image is represented by an array containing integers ranging from 0 to 255. We rewrite the integers larger (smaller) than 255/2 as 1 (-1) and reduce the image to a 14×14 array with a pooling process before training. The corresponding handwritten digit is shown as the insert of Fig. 4g. Then, a BNN with 196 input nodes, 14 hidden nodes, and 1 output node is trained. The output $\bar{x}_1^3$ is desired to be -1 (1) when the input handwritten digit is 0 (1). The training process is shown in Fig. 4g. The error means the number of handwritten digits failed to be distinguished in the training set (or testing set). The errors for the training and testing sets gradually reduce and

become 1 after about 170 training steps. Considering the equivalence of the BNN and MBNN mentioned in the main text, the training method can serve as a strong tool to design intelligent deformation input-output relationships that can even adapt to unseen conditions by its ability of generalization.

Except for the MBNN shown in the main text, we have also constructed an MBNN with different network structures. An example is shown in Supplementary Figure 5a, with the corresponding schematic diagram and experiment setup given in Supplementary Figure 5b and c, respectively. Using the training method mentioned before, the MBNN can be reprogrammed to fulfill all 16 logic gates with two inputs and one output (i.e., AND, OR, NOR, XOR gates, and so on) as shown in Supplementary Movie 3. This experiment again demonstrates the equivalence between the BNN and MBNN.

Supplementary Note 5: Construction of the mechanical self-learning perceptron with *n*+1 inputs

A Rosenblatt perceptron with $n+1$ inputs is shown in Supplementary Figure 6a. Its forward propagation procedure is:

$$y=\varepsilon\left(\tilde{\boldsymbol{w}}^T\boldsymbol{x}\text{-}1\right)$$
$$\tilde{\boldsymbol{w}}=\left[w_0,w_1,\ldots,w_n\right]^T,\boldsymbol{x}=\left[x_0,x_1,\ldots,x_n\right]^T \tag{1}$$

where y is the output, x_i is the input, w_i is the weight, $x_i\in \mathrm{B}$ and $w_i\in \mathrm{R}$, i=0, 1, 2,…, n . The weights of the backward propagation are updated as:

$$\tilde{w}_i=\tilde{w}_i+\mathrm{sign}\left(y_t-y\right)x_i\mathrm{d}\tilde{w} \tag{2}$$

where y_t is the target output, and $d\tilde{w}$ is the updating increment. The corresponding mechanical self-learning perceptron model is shown in Supplementary Figure 6B. Its forward propagation procedure represented by the black interaction symbols is the same as Eq. (1). The weights can be written as:

$$\tilde{w}_i=\gamma_i+\alpha_i\sum_{j=1}^{m}x_{ij},i=0,1,2,3... \tag{3}$$

In the backward propagation procedure denoted by the brown interaction symbols, the memory elements evolve as:

$$\begin{cases} x_{i1} = x_{im}, x_{i2} = x_{i1}, \ldots, x_{im} = x_{i(m-1)}, c = 1 \\ x_{i1} = y_t, x_{i2} = x_{i1}, \ldots, x_{im} = x_{i(m-1)}, c = 0 \end{cases} \tag{4}$$
$$i = 0, 1, 2, \ldots, n$$

Thus, the weights are updated as follows:

$$\tilde{w}_i = \tilde{w}_i + \alpha_i \left| y_t - x_{im} \right| \operatorname{sign}\left(y_t - y \right) x_i \tag{5}$$

Equation (5) is a variant version of (2) where $d\tilde{w} = \alpha_i \left| y_t - x_{im} \right|$. Though the update increments vary for different weights in different updating steps, we have verified that the weights can still converge by selecting a small α_i , large m , and randomly setting the initial value of x_{ij} .

The verification results are shown as three typical cases listed in Supplementary Figure 6c and d. For cases 1, 2, and 3, n=9, 19, and 29, while 300 target input-output pairs ($\boldsymbol{x}$, y_t) are randomly selected and can form two node sets defined by the target output y_t on both sides of the hyperplane described by equations (6-8) respectively.

$$\begin{aligned} &-13.15x_0 + 15.175x_1 + 15.825x_2 - 13.75x_3 + 17.4x_4 \\ &+22.45x_5 + 10.525x_6 - 3.8x_7 + 8.7x_8 - 24.525x_9 = 1 \end{aligned} \tag{6}$$

$$\begin{aligned} &3.15x_0 - 1.9x_1 + 6.8x_2 - 21.375x_3 - 2.075x_4 - 24.175x_5 - 16.325x_6 \\ &-0.05x_7 + 2.475x_8 + 14.25x_9 + 0.2x_{10} - 17.925x_{11} + 14.175x_{12} \\ &-24.2x_{13} - 17.85x_{14} - 5.2x_{15} - 14.025x_{16} + 20x_{17} - 18.025x_{18} + 7.2x_{19} = 1 \end{aligned} \tag{7}$$

$$\begin{aligned} &17.65x_0 - 12.675x_1 - 3.45x_2 - 16.3x_3 - 23.4x_4 + 8.9x_5 + 14.625x_6 \\ &-1.35x_7 + 16.5x_8 - 3.875x_9 - 20.15x_{10} - 5.625x_{11} - 10.625x_{12} \\ &-7.625x_{13} - 11.45x_{14} + 14.6x_{15} - 22.125x_{16} - 20.95x_{17} + 3.15x_{18} + 9.95x_{19} \\ &+21.8x_{20} + 2.075x_{21} + 6.55x_{22} - 24.225x_{23} - 9.25x_{24} + 19.3x_{25} + 8.625x_{26} \\ &+16.625x_{27} + 17.625x_{28} - 10.075x_{29} = 1 \end{aligned} \tag{8}$$

We set $\alpha_i = 1/40$, $\gamma_i = -25$, $m = 2000$ for all three cases. The error is defined as the number of outputs that fail to meet the target output and its evolution is shown in Fig. 5f. The mechanical self-learning perceptron acquires all the target output after about 3500 training steps. As another example to show the updating process, the evolution of

$\tilde{w}_0$ is given in Fig. 5g. These examples show the generality of the mechanical self-learning perceptron.

List of supplementary figures

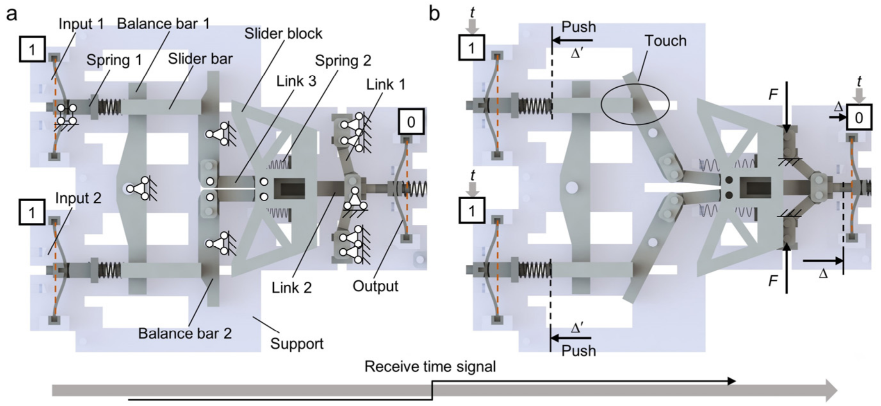

Supplementary Figure 1. Computing process of the mechanical XNOR structure for input (1, 1). a Before receiving the time signal. **b** After receiving the time signal.

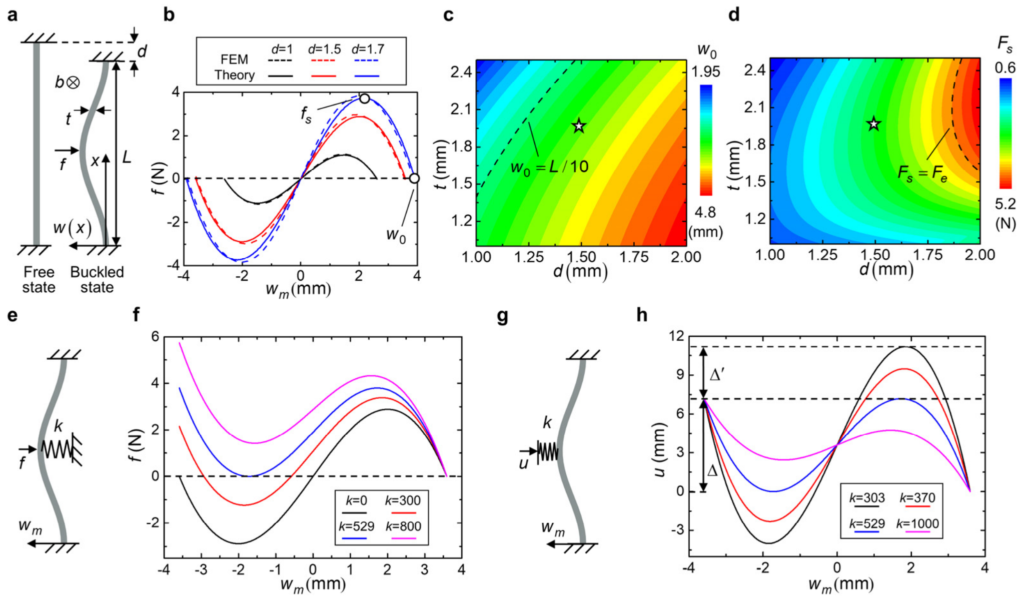

Supplementary Figure 2 Mechanics of the buckled beam without and with a spring. **a** Geometry and notation for the buckled beam. **b** FEM and theoretical results of the force-deflection curves (f-w_m) of the buckled beam in a. **c** Contour of w_0 in the d-t space. **d** Contour of fs in the d-t space. **e** A buckled beam connected to a spring with a fixed end. **f** Theoretical results of the f-w_m relationship for the buckled beam in e. **g** A buckled beam is compressed via a connected spring. **h** Theoretical results of the u-w_m relationship for the buckled beam in g.

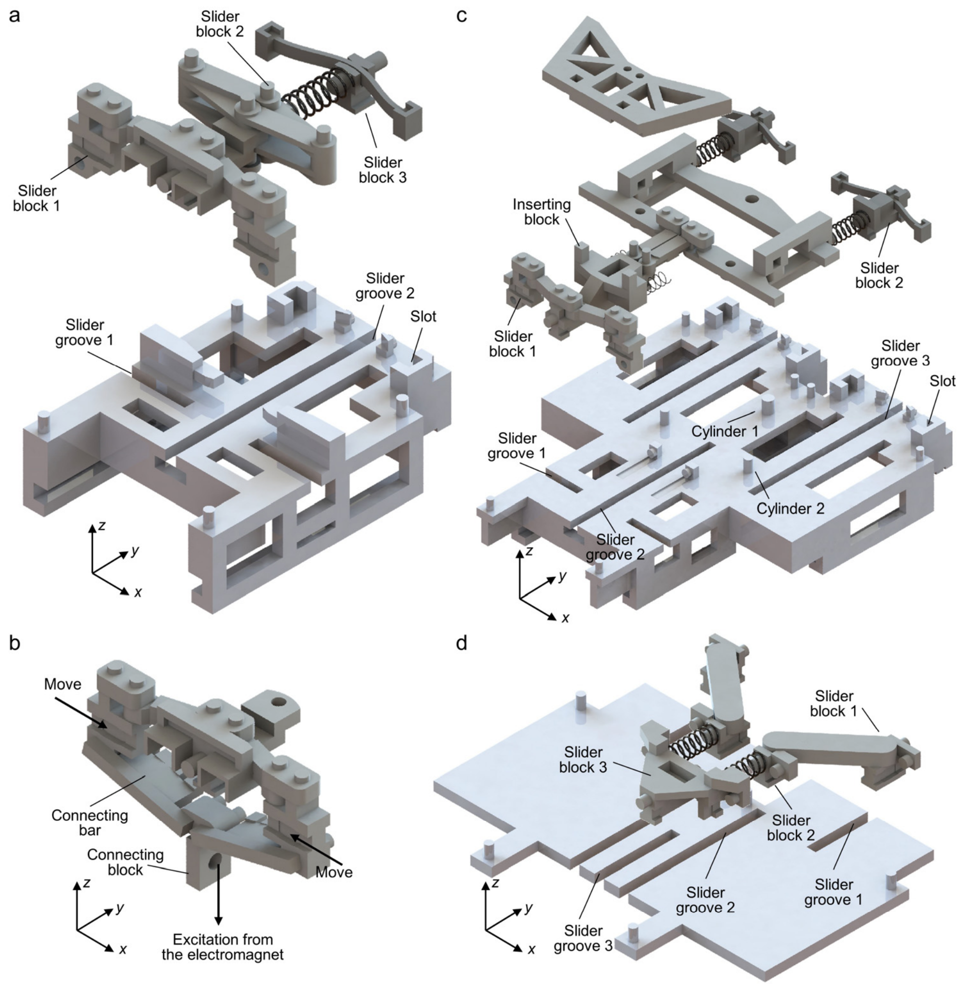

Supplementary Figure 3 Assembling details of the basic interaction structures. a Assembling of the mechanical shift register. **b** Method to impose time signal. **c** Assembling of the mechanical XNOR structure. **d** Assembling of the mechanical perceptron.

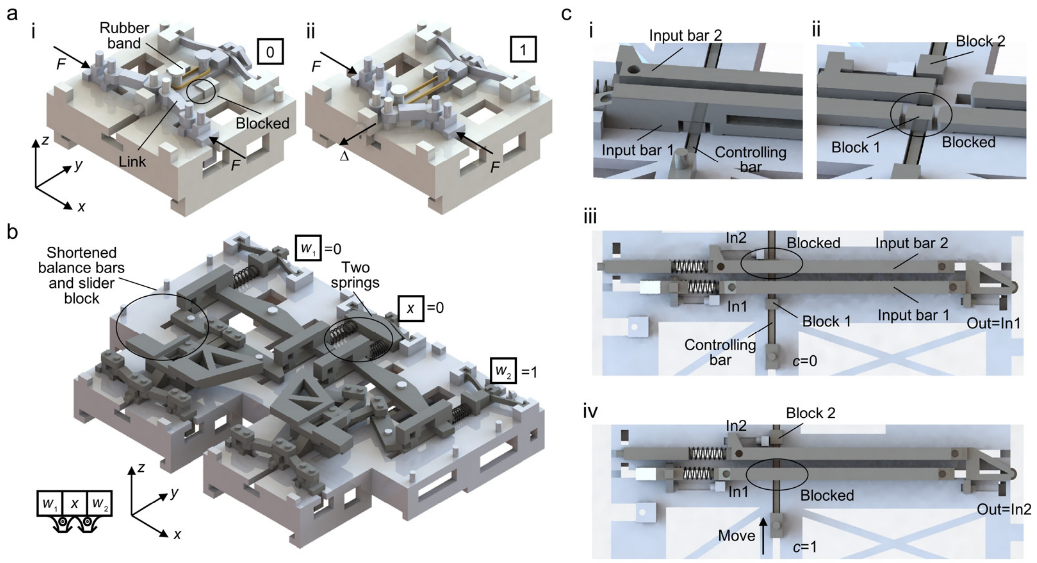

Supplementary Figure 4 Design of other structures required for computing. **a** Structure that stores the bias. For (i), the bias is 0. For (ii), the bias is 1. **b** A variant design for the mechanical XNOR operation. **c** Design of the data selector. (i) shows the main components. (ii) is a sectional view of (i). (iii) (iv) Computing process of the data selector. For (iii), the output is In1 when c=0. For (iv), the output is In2 when c=1.

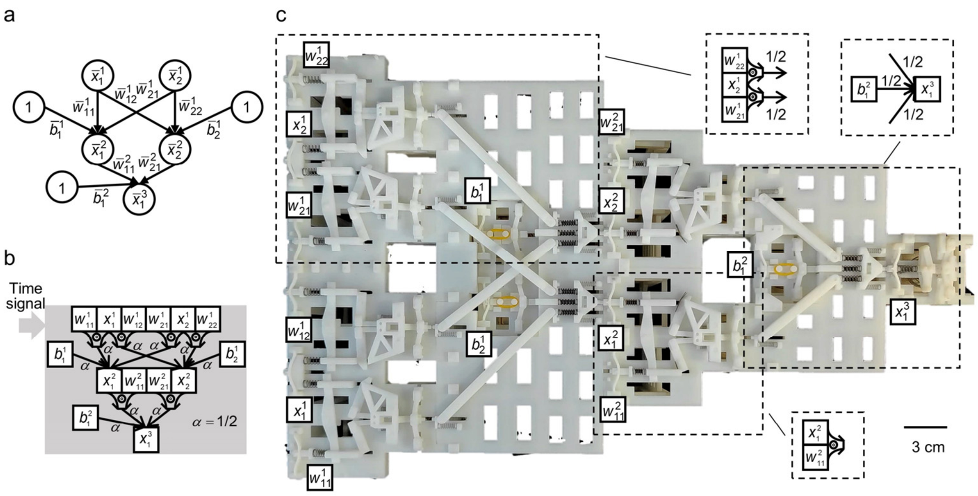

Supplementary Figure 5 A reprogrammable mechanical binary neural network to realize all 16 logic gates of two inputs and one output. a The corresponding binary neural network. **b** The schematic diagram and **c** the experiment setup of the mechanical binary neural network.

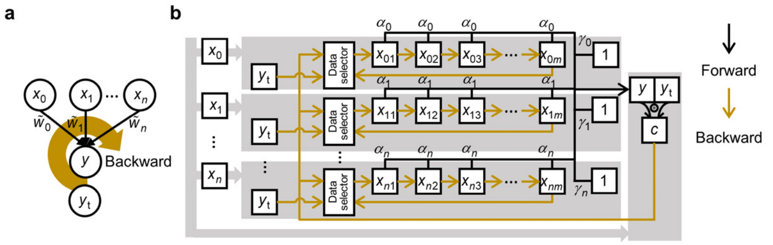

Supplementary Figure 6 A mechanical self-learning perceptron with *n*+1 inputs. **a** A perceptron model with *n*+1 inputs. **b** The schematic diagram of the mechanical self-learning perceptron.
