# Supplementary material for: In-memory mechanical computing
Source: Nat Commun. 2023 Aug 25;14:5204. doi: 10.1038/s41467-023-40989-1 (PMC10457397; doi:10.1038/s41467-023-40989-1)
Supplement: Supplementary file 3 — Description of Additional Supplementary Files [file 41467_2023_40989_MOESM3_ESM.pdf]

Description of Additional Supplementary Files

File Name: Supplementary Movie 1

Description: A mechanical binary neural network with five nodes in the input layers, two nodes in the hidden layer, and one node in the output layer. The network can be employed to evaluate the parity of the input Morse code numbers 0–9, and then reprogrammed to determine whether an input Morse code number belongs to the section [4, 8].

(1) Evaluate the parity of Morse code numbers 0–9

(2) Determine whether a Morse code number belongs to [4, 8]

File Name: Supplementary Movie 2

Description: A mechanical binary neural network with two nodes in the input layers, two nodes in the hidden layer, and one node in the output layer. In total, there are 16 logic gates for the cases of two inputs and one output (such as AND, OR, XOR, and so on). The network is reprogrammed to realize all these 16 logic gates.

File Name: Supplementary Movie 3

Description: The mechanical self-learning perceptron. The learning procedure for all possible target input-output relationships are shown, i.e.,

(1) Input x=0, output y=0, and input x=1, output y=0.

(2) Input x=0, output y=0, and input x=1, output y=1.

(3) Input x=0, output y=1, and input x=1, output y=0.

(4) Input x=0, output y=1, and input x=1, output y=1.
